# Nature-Inspired Solutions: Biomimetic Materials and Adaptive Devices for Precision Urinary Oncology

**DOI:** 10.3390/cancers18091429

**Published:** 2026-04-30

**Authors:** Chunlian Zhong, Lifeng Yin, Michael Hung, Shanshan Yao, Menghuan Tang, Zhaoqing Cong

**Affiliations:** 1 Department of Urology, Stony Brook University, Stony Brook, NY 11794, USA; chunlian.zhong@stonybrookmedicine.edu (C.Z.); lifeng.yin@stonybrook.edu (L.Y.);; 2 Stony Brook Cancer Center, Renaissance School of Medicine, Stony Brook University, Stony Brook, NY 11794, USA; 3 Department of Mechanical Engineering, Stony Brook University, Stony Brook, NY 11794, USA; shanshan.yao@stonybrook.edu; 4 Department of Biochemistry and Molecular Medicine, University of California, Davis, CA 95817, USA

**Keywords:** urinary cancer, urothelial barriers, intravesical drug delivery, biochemical biomimicry, structure and motility biomimicry

## Abstract

Urinary cancers present a severe clinical challenge due to high recurrence rates. Standard intravesical therapies suffer from limited efficacy because of the urinary tract’s robust physiological defenses, namely, the dynamic washout effect during voiding and highly restrictive urothelial barriers, such as the anti-adhesive glycosaminoglycan layer and intercellular tight junctions. This review aims to explore how biomimetic engineering can overcome these obstacles by transitioning drug delivery from passive carriers to active, nature-inspired systems. We conducted a comprehensive review of the recent literature focusing on biomimetic strategies for intravesical drug delivery and urinary cancer theranostics. The analyzed approaches are categorized into chemical biomimicry (such as adhesion and camouflage) and structural/functional biomimicry (including adaptive devices and microrobots). Biomimetic strategies significantly enhance targeted drug retention and tissue penetration. Chemical biomimicry, utilizing mussel-inspired catechol chemistry and cell membrane camouflage, effectively bypasses the urothelial anti-adhesive defenses and reduces the immune clearance. Structural and functional biomimicry, such as naturally derived carriers and actively propelled magnetic or biohybrid microrobots, enables the precise spatial localization and controlled payload release in dynamic fluid environments. Furthermore, lab-on-a-chip technologies and patient-derived organoids (PDOs) offer scalable platforms for screening cargo-specific efficacies and tailoring treatments, providing a crucial bridge to personalized precision medicine. Integrating nature-inspired designs with advanced nanotechnologies provides a highly promising pathway with which to overcome the mechanical and biological barriers of the urinary tract. These biomimetic innovations hold the potential to shift the therapeutic paradigm for urinary oncology, paving the way for more efficient, targeted, and personalized precision medicine.

## Simple Summary

Treating urinary cancers is highly challenging because the body’s natural defenses, such as frequent urination and strong cellular barriers, quickly wash away or block conventional medicines. To solve this problem, we explored new drug delivery technologies inspired by nature. For example, by studying how marine mussels firmly attach to wet rocks or how certain cells camouflage themselves to hide from the immune system, scientists can design “smart” materials that stick better to the urinary tract. We review these nature-inspired strategies, including microscopic robots that can actively swim through fluids to deliver drugs directly to tumors. By summarizing these biomimetic advancements, this review aims to guide researchers in developing more effective, longer-lasting, and highly targeted therapies. Ultimately, these biological innovations could transform how we treat urinary cancers, leading to better patient care and personalized medicine.

## 1. Introduction

### 1.1. The Clinical Challenge: Cancer Treatment in the Actively Flushing Urinary Tract System

Urinary cancers, including bladder cancers (BCs), upper tract urothelial cancers (ureteric and renal pelvic cancers, UTUCs), and urethral cancers are the second most common cancer and contribute to the fifth most common cancer-related death in males [1]. The most common form of urinary cancers is urothelial cancers, classified as a non-muscle-invasive location in the mucosa or submucosal connective tissue and muscle-invasive cancers invading into the detrusor muscle layer [2]. Statistically, approximately 169,700 new urinary cancer cases will be diagnosed and 34,400 deaths are projected to occur in the United States in 2026, according to about 465 new cases and 94 deaths each day. BCs have the highest incidence and mortality [1]. Around 75% of patients were diagnosed with non-muscle-invasive bladder cancers (NMIBCs) [3]. Surgery resection combined with intravesical therapy, such as BCG vaccine [4], chemotherapy [5], gene therapy [6], and immune-checkpoint inhibitor (ICI) therapy [7], is a standard management for NMIBC [8], which is responsible for about 90% of overall survival [3]. However, the recurrence and/or progress of NMIBC are frequent, 31–78% and 1–45% within five years, respectively [3]. Alarmingly, untreated patients with high-risk NMIBC could further progress to muscle-invasive bladder cancers (MIBCs) with five years of 40–80%, which is associated with a much lower median overall survival of 15 months [9].

BCG is recommended as the first-line therapy for NMIBC; however, the ongoing shortages make it not suitable for some patients. For example, BCG-induced proinflammatory cytokines cause flu-like symptoms in patients [10]. Dysuria induced by the bladder instillation of BCG was also observed in 60% of patients [11]. Additionally, BCG treatment is not effective for some patients with an intolerance or resistance [12]. Radical cystectomy with urinary diversion is a standard care for these patients, but this approach is commonly linked to notable morbidity and lifestyle modification [2]. Alternatively, intravesical chemotherapy (e.g., mitomycin C and gemcitabine) [13], immunotherapy (e.g., pembrolizumab) [6], and gene therapy (e.g., nadofaragene firadenovec) [14] are better options. Typically, chemotherapeutic agents generally require repeated weekly or monthly intravesical instillations to maintain the effective chemotherapeutic concentrations [15]. In contrast, a single dose of intravesical gene therapy can bring out a therapeutic response, and multiple doses are essential for a favorable long-term prognosis. For example, 55 of 103 patients with BCG-unresponsive NMIBC showed a complete response to a single dose of nadofaragene firadenovec instillations; a repeat dose at months 3, 6, and 9 maintained this response through 12 months in 25 of 55 patients [6]. Unlike intravesical instillation, immunotherapy is often administrated through intravenous injections repeatedly. Pembrolizumab monotherapy intravenously every 3 weeks for up to 24 months showed significant antitumor activity in NMIBC with or without carcinoma in situ [16]. For UTUCs, contributing to only 5–10% of all urinary cancer [1], the clinical management is different due to the distinct pathological features of bladder cancer. While radical nephroureterectomy remains a standard method of care, it is frequently associated with a poor prognosis and high recurrence risk [17,18]. The upper urinary tract instillation of chemotherapy (e.g., mitomycin C, cisplatin, and gemcitabine) or immunotherapy agents (e.g., pembrolizumab and atezolizumab) provide an alternative consideration [17]. Similarly, for rare urethral cancers, postoperative chemotherapy (e.g., cisplatin, 5-fluorouracil, and mitomycin C) demonstrates significant survival advantages for advanced patients [19]. Therefore, intravesical instillations of chemotherapy and gene therapy provide a potential chance for favorable urinary cancer treatment. However, the washout-effect-induced short drug retention in the urinary tract and passive-diffusion-associated low drug concentration substantially reduces drug efficacy. Therefore, the development of a novel drug delivery system is essential for urinary cancer treatment.

### 1.2. The Biological Barrier

**Figure 1:**
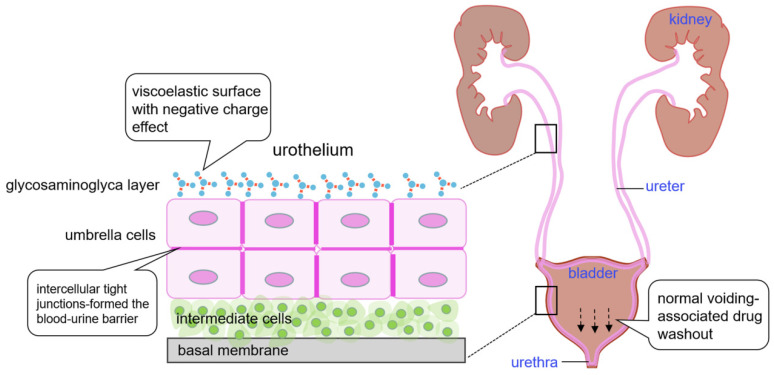
Mechanical and biological barriers of the urinary tract. The urothelium comprises basal membrane, intermediate cells, and superficial umbrella cell layers, which are covered by negatively charged glycosaminoglyca layer. These anatomical characteristics combined with washout effect during voiding period inhibit drug absorption and retention in the urothelium. Gray: basal membrane; light green: intermediate cells; light purple: umbrella cells; dark purple: intercellular tight junctions; gray–blue: glycosaminoglyca layer.

In addition to the normal voiding-associated washout effect, the anatomy and physiology characteristics of the urinary tract limit drug delivery (Figure 1). The urothelium lines the luminal surface of the urinary tract, extending from the renal pelvis through the ureters and bladder to the proximal urethra, which consists of basal, intermediate, and superficial umbrella cell layers, the latter of which is covered by around 90% of urothelial plaques assembled from uroplakin protein heterodimers that contribute to urinary barrier function [20]. This rigid architecture combined with a high cholesterol content and uroplakin–cytoskeleton complex form a formidable physical shield for drug absorption. The glycosaminoglyca (GAG) layer located at the surface of superficial umbrella cells is another main factor in maintaining urinary barrier function. The anionic properties of chondroitin sulfate and hyaluronic acid within the GAG layer form the first biological defense line against the adhesion and penetration of bacteria and harmful substances within the urine [21]. The GAG layer not only physically blocks the drug’s passive diffusion before it reaches the superficial umbrella cell membrane but also provides a chemical repulsion for drug adhesion.

Additionally, intercellular tight junctions (TJs) of umbrella cells form the blood–urine barrier, limiting the transepithelial transport of water and drug molecules [22]. In contrast to other tissues, umbrella cells have much tighter TJs, in which cytoplasmic plaque proteins such as zonular occludens (ZO) proteins anchor junctional transmembrane proteins including occludin, claudins, and junctional adhesion molecules to the actin cytoskeleton, establishing a completely enclosing barrier around each cell [23]. These TJs block the paracellular pathway and effectively restrict drug transport between the apical and basolateral membrane of umbrella cells. Urine is another factor that compromises drug absorption in the urothelium. Apart from the normal urine-voiding-mediated drug washout, urine biochemical components influence drug stability. Urine, a main metabolism product, consists of a large amount of water, urea, toxins, electrolytes, and other substances. Urinary pH is usually acidic or weakly acidic, with a pH range of 5.3 to 6.5 [24]. Not only do these biochemical components alter the urothelial permeability through modulating ion transport systems [25,26], but the acidic environment can also accelerate drug degradation and weaken drug efficacy [27]. In addition, changes in the urine biochemistry can aid in diagnosing the disease stage and evaluate the therapeutic efficacy [28]. Therefore, a therapeutic paradigm shift is essential in order to overcome these mechanical and biological barriers of the urothelium and urinary interference.

### 1.3. The Biomimetic Paradigm Shift

To overcome these urothelial barriers, several drug delivery methods have been developed. For example, chemo-hyperthermia [29] and electromotive drug administration [30] aim to stimulate urothelial permeability to improve drug absorption. Mechanical delivery provides a sustained drug release platform that effectively mitigates the urinary washout effect, thereby maximizing the antitumor efficacy in MIBC [31]. Drug complexes integrate functional components such as hydrogels [32], nanoparticles [33], or hyaluronidase [34] to enhance the drug contact time with the urothelium. However, one of the main issues is the safety of these delivery methods, which needs to be further assessed by clinical trials. As an alternative, the development of biomimicry offers a novel strategy for intravesical drug delivery.

Biomimicry is an interdisciplinary field that emulates the structure and composition in nature to create a better performance beyond natural function [35,36] and has made notable strides in various fields, including environmental protection, energy revitalization, biomedicine, ecological restoration, and agronomic interventions [37]. For example, in the biomedical field, mussel-inspired hydrogels significantly improve the biological adhesion and drug release of the transdermal drug delivery system [38]. Biomimetic microrobots that mimic the structures and functions of pathogens or mammalian cells [39] leverage natural tropism to actively target tumor cells for therapeutic agent delivery. These biomimetic applications evolve drug delivery patterns from “inert, passive implants” to “active, life-like systems”, providing the potential to increase the drug concentration in the urothelium and shift the therapeutic paradigm of urinary cancer. In this review, we describe the mechanical and biological barriers for urinary cancer treatment and outline the biomimetic strategies for intravesical drug therapy, future directions, and challenges.

For the literature review, our search strategy and study selection were broadly guided by the principles of the PRISMA framework [40]. We systematically searched the PubMed database through 2026 using the keywords “urinary cancers”, “drug delivery”, “urinary tract system”, “biomimetic materials”, “microrobots”, “magnetic nanoparticles”, “lab-on-a-chip”, and “tumor organoids” in the PubMed database. We identified and selected articles that provided evidence for the biomimetic applications in cancer treatment. The rationality and preclinical evidence of the development of biomimicry in the drug delivery system and their effects on urinary cancer treatment were summarized and compared.

## 2. Chemical Biomimicry: Materials for Adhesion, Camouflage, and Penetration

### 2.1. Mussel-Inspired Adhesion for Enhanced Drug Retention

**Figure 2:**
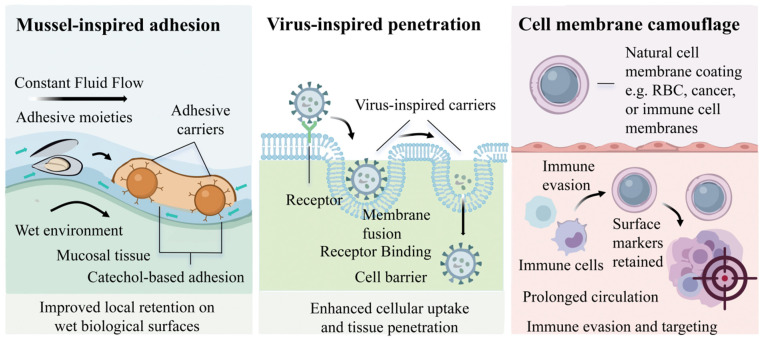
Representative biomimetic interfacial strategies for therapeutic delivery, including mussel-inspired adhesion for wet surface retention, virus-inspired design for cellular entry and barrier crossing, and cell membrane camouflage for immune evasion and targeted delivery.

Drug delivery in the urinary tract remains challenging because continuous fluid flow can rapidly remove therapeutic agents from the treatment site. This washout shortens the local residence time and weakens the therapeutic efficacy [41]. Most conventional delivery systems rely on passive diffusion or weak interactions with tissue surfaces. As a result, they often fail to maintain stable retention at the target site [41]. To address this problem, researchers have increasingly turned to biomimetic adhesion strategies derived from natural systems [42,43]. Among them, mussel-inspired adhesion has attracted particular attention because it can maintain a strong adhesive performance in wet physiological environments (Figure 2) [44]. Recent reviews further indicate that catechol-based materials, especially polydopamine and injectable adhesive hydrogels, have become a major direction in biomedical adhesion and drug delivery research [45,46].

Marine mussels can attach firmly to diverse surfaces under dynamic aqueous conditions. This ability mainly arises from adhesive proteins rich in catechol groups, especially DOPA. The catechol chemistry supports several interfacial interactions at once, including hydrogen bonding, covalent interactions, and metal coordination [42]. Because these interactions act together, mussels can remain stably attached even in flowing water. This feature has made mussels an important biomimetic model for biomedical material design. Among the artificial systems derived from this principle, polydopamine is the most representative [46,47]. It can form uniform coatings on various substrates under mild conditions and can then be used for drug loading, surface modification, and the regulation of biological interactions [48]. Menichetti et al. further emphasized that the size, morphology, and surface charge of polydopamine nanosystems strongly influence the circulation, uptake, loading, and release behavior [47].

Recent studies further illustrate the translational value of this strategy. Dou et al. summarized the use of mussel-inspired injectable adhesive hydrogels in wound repair, hemostasis, bone repair, surface coatings, sensing, and drug delivery [46]. More directly relevant to local urinary delivery, Wang et al. developed a lactobacillus polydopamine system for bladder therapy and evaluated it using bladder spheroids, rat models, and urinary microbiome analysis [49]. Their results suggest that polydopamine-based surface modification can improve local distribution and tissue penetration in bladder-related delivery. In parallel, related catechol-based systems have also been explored in vaccine delivery [50], antibacterial titanium alloy screws [51], underwater adhesive hydrogels [52], antibacterial polymer coatings [53], zirconia implants with improved bone integration [54], antifouling loose nanofiltration membranes [55], tannic acid functionalized coatings for colonic delivery [56], and polysaccharide-based multifunctional hydrogels [57]. Taken together, these studies extend the significance of mussel-inspired adhesion from general wet adhesion to clinically relevant local retention and site-specific delivery.

Overall, mussel-inspired adhesive systems offer several clear advantages, including strong wet adhesion, broad surface adaptability, and generally good biocompatibility. In the urinary tract, however, improved adhesion should not be treated as an isolated advantage, because local retention must be balanced against clearance, material degradation, urothelial tolerance, and the risk of persistent adhesion or inflammation. More work is still needed to regulate the adhesion strength, material degradation, and long-term safety with greater precision.

A recent bladder-specific study further illustrates this point. Zheng et al. reported an intravesical tumor-selective mucoadhesive hydrogel for bladder cancer in a murine model, showing that mucoadhesion can be coupled to tumor-selective local chemotherapy rather than indiscriminate prolonged residence [58]. This type of design is more clinically relevant for intravesical therapy because it begins to address the practical balance among retention, local activity, and eventual clearance in the bladder. Importantly, this balance may also vary with the cargo class. Prolonged mucosal contact may be beneficial for small-molecule intravesical chemotherapy, whereas biologics, nucleic-acid cargoes, or sustained-release systems may impose different requirements for clearance, degradation, and local safety.

### 2.2. Viral-Inspired Penetration Strategies

Biological barriers such as epithelial layers, mucus-rich luminal interfaces, and intracellular membranes strongly restrict drug penetration, especially for macromolecules, nucleic acid cargoes, and some nanoparticle systems [59,60]. By contrast, viruses have evolved highly efficient mechanisms for recognition, entry, membrane fusion, and intracellular delivery over long periods of evolution [61]. For this reason, viral entry pathways provide an attractive model for biomimetic drug delivery design (Figure 2). Current studies do not simply copy viruses directly. Instead, they borrow key features such as membrane fusion, receptor recognition, and barrier crossing strategies, then integrate them into safer engineered carriers [62]. In this sense, viral inspiration is now less about literal imitation and more about extracting functional design rules from viral behavior.

A representative example comes from the study by Wang et al., who designed biomimetic nanovesicles containing both fusogenic components and targeting moieties [62]. The goal of this design was to improve cytosolic delivery efficiency and targeting specificity, which are also two major strengths of viral infection. The importance of this work lies in the fact that it translated two core viral principles, membrane fusion and selective recognition, into designable features within a nonviral delivery system. For that reason, this study aligns closely with the theme of virus-inspired penetration rather than general nanocarrier functionalization. Even so, for urinary oncology, the relevance of this strategy becomes more convincing when it is linked to bladder-specific barriers and to a clearly defined therapeutic cargo.

More direct urinary evidence is now available. Zheng et al. developed R11-modified tumor cell membrane nanovesicle-camouflaged nanoparticles for the intravesical chemotherapy of bladder cancer and showed that this design improved mucus penetration, homologous tumor targeting, and local therapeutic efficacy in orthotopic models [63]. The relevance of this study is that it does not treat penetration as an abstract material property. Rather, it addresses a specific intravesical problem, namely, how to deliver a chemotherapeutic payload across the mucus and urothelial barrier while reducing off-target exposure to normal urothelial cells. In this setting, penetration is discussed in relation to a defined cargo class, intravesical chemotherapy, rather than as a generic improvement in drug delivery.

A second bladder-specific example further clarifies that penetration requirements depend on the therapeutic cargo. Liu et al. reported an intravesical ICG@R11-CeO_2_ nanozyme platform for photodynamic therapy, in which mucoadhesion, mucus penetration, tumor selectivity, and local oxygen modulation were integrated within the same system [64]. In this case, the goal was not simply deeper penetration, but the selective delivery of a photosensitizer to orthotopic bladder tumors under conditions that also improved PDT responsiveness. This distinction is important, because the design requirements for intravesical photodynamic therapy differ from those for conventional cytotoxic chemotherapy. By contrast, several non-urologic examples, including hybrid biomimetic nanovesicles developed for glioma treatment and blood–brain barrier transport, remain conceptually informative because they show how membrane fusion and receptor-guided entry can be converted into engineering rules for difficult tissue barriers [59,65].

Recent studies on viral-like particles also help define the conceptual scope of this section more clearly. Travassos et al. noted that viral-like particles combine structural stability, biocompatibility, scalable preparation, and the ability to mimic viral organization while lacking viral genetic material [66]. This point matters because it allows researchers to retain many advantages of viral design without introducing infectivity. At the same time, recent reviews on biomimetic nanocarriers for intracellular protein delivery and on non-antibody toxin conjugates further indicate that understanding membrane entry and intracellular trafficking remains central to efficient delivery design [67]. For urinary oncology, however, the practical value of these concepts depends on whether they can be adapted to the specific constraints of intravesical therapy, including the rapid washout, the GAG layer, the limited residence time, and local urothelial tolerance. Taken together, virus-inspired penetration is no longer only a conceptual analogy. It has become a practical design strategy for improving cellular entry, intracellular delivery, and barrier crossing [68]. Its translational value in urinary oncology is strongest when discussed in relation to defined therapeutic cargoes and bladder-specific evidence rather than as a generic enhancement in drug delivery.

### 2.3. Cell Membrane Camouflage for Immune Evasion and Targeting

Rapid in vivo clearance remains one of the main limitations of synthetic nanoparticles. Many carriers are removed by the reticuloendothelial system before they reach diseased tissue, while others are taken up nonspecifically by healthy cells [69]. Cell membrane camouflage has therefore attracted increasing attention because it allows artificial nanoparticle cores to inherit biologically meaningful features from natural cell surfaces (Figure 2) [69,70]. Recent reviews show that cell-membrane-coated nanoparticles have become an important biomimetic platform for improving immune evasion, prolonging circulation, and enhancing targeting, especially in cancer immunotherapy and related delivery settings [70]. From a broader translational perspective, this strategy can also be understood as part of the wider development of tumor-targeting biomimetic drug delivery systems, in which endogenous components are used to reduce immune clearance and improve targeting accuracy [71]. For intravesical therapy, this strategy is also relevant because different cargo classes do not face the same delivery problem. Small-molecule chemotherapy may benefit mainly from improved local retention and selective uptake, whereas immune agonists and other biologically active payloads depend more strongly on precise targeting and controlled local exposure.

The advantage of this strategy does not lie in the membrane structure alone. Membrane lipids, membrane proteins, and cell-specific surface markers all contribute to immune recognition and tissue interaction. Once these features are transferred onto nanoparticle surfaces, the resulting carriers behave more like endogenous biological entities. This also explains why different membrane sources offer different benefits. Red blood cell membranes are commonly used to prolong circulation, cancer cell membranes support homologous targeting, and immune cell membranes are more suitable for inflammation-related or tumor-associated delivery [71]. In this sense, membrane camouflage is not simply a stealth coating. It is a way of transferring biologically evolved interfacial information to synthetic carriers.

More recent work in bladder cancer shows that membrane camouflage is moving beyond passive immune evasion toward programmable targeting. Deng et al. developed genetically engineered T-cell-membrane-camouflaged nanoparticles in which Tim-3 overexpression enhanced tumor recognition through the Tim-3/Galectin-9 axis [72]. In this design, membrane engineering was used not only to prolong persistence or reduce immune clearance, but also to install a defined targeting mechanism in bladder cancer. This shift is important because it moves membrane camouflage beyond passive stealth and toward mechanism-guided targeting.

The translational potential of this strategy has been further expanded by engineering studies. Park et al. reported genetically engineered cell-membrane-coated nanoparticles for the targeted delivery of dexamethasone to inflamed lung tissue [73]. The source cells were modified to express very late antigen-4 (VLA 4), and the resulting membrane-coated nanoparticles showed a higher affinity for the inflamed endothelium expressing vascular cell adhesion molecule-1 (VCAM 1), along with improved delivery and therapeutic effects in vivo. This study remains useful as an engineering precedent showing that membrane camouflage can be actively optimized through genetic modification, although it should not be interpreted as direct urinary-oncology evidence.

A more directly relevant urinary example was reported by Wu et al., who used genetically engineered hybrid-membrane-coated nanoparticles to enhance dendritic-cell activation for bladder cancer immunotherapy [74]. Their system showed significant antitumor effects in multiple preclinical settings, including subcutaneous, orthotopic, and distant bladder tumor models. This finding suggests that membrane engineering can support not only immune evasion but also the active remodeling of local antitumor immunity in bladder-relevant models.

Taken together, cell membrane camouflage improves delivery through two closely related routes. First, it reduces immune clearance and extends exposure time [75]. Second, it introduces disease-relevant recognition cues that enhance targeting ability [72]. Its relevance to urinary oncology becomes much stronger when the discussion is tied to specific cargo classes and bladder-specific models, particularly for immunotherapy-oriented payloads and engineered targeting systems [74]. Future work still needs more robust preparation methods, clearer quality control standards, and longer-term safety evaluation. Even so, the field now appears to be moving beyond simple membrane transfer toward engineered membrane systems with tunable biological functions [74].

## 3. Structural and Functional Biomimicry: Adaptive Devices and Microrobots

### 3.1. Natural Carriers: Utilizing Nature’s Geometries

**Figure 3:**
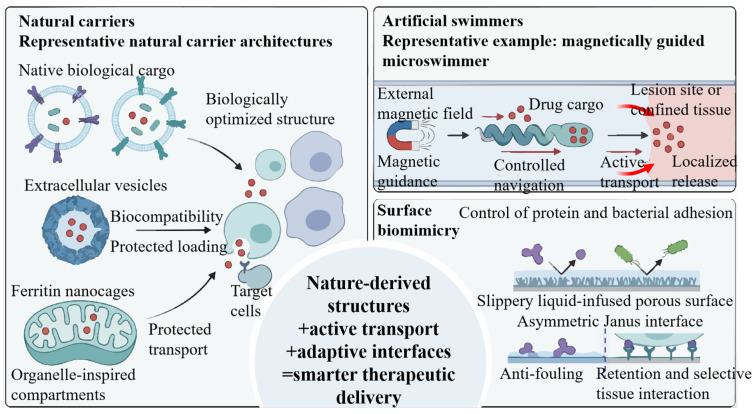
Representative biomimetic platforms for smarter therapeutic delivery, including natural carriers for protected transport, artificial swimmers for active navigation and localized release, and surface biomimicry for adaptive interfacial regulation.

Natural carriers continue to attract interest not only because they are generally biocompatible, but also because their structures have already been optimized by biology for transport, protection, and molecular communication (Figure 3) [76]. Exosomes, ferritin, viruses, and cellular organelles [69,77] are all naturally organized systems that can support cargo loading, stabilization, and biological interaction in different ways [78]. Compared with many synthetic carriers, these platforms are often better adapted to biological environments and may retain useful features for recognition [68,69], trafficking, and payload protection. Recent reviews further suggest that extracellular vesicles and organelle-based systems are becoming central themes in therapeutic cargo delivery research [69,79]. In the urinary setting, the value of these carriers depends less on their natural origin alone and more on whether they can preserve fragile cargoes under repeated dilution, a brief dwell time, and the urothelial barrier.

Among these systems, extracellular vesicles are the most visible example. They are heterogeneous membrane-bound carriers that can transport proteins, lipids, and nucleic acids while maintaining a low immunogenicity and efficient cellular uptake. Their importance lies not simply in their natural origin, but in the fact that they already function as biologically competent delivery structures. In other words, extracellular vesicles are not passive shells that become useful only after engineering. Rather, they are pre-existing functional platforms whose native architecture already supports transport and intercellular exchange [79]. This point is especially relevant for protein, nucleic acid, and other labile cargoes, for which structural protection may be as important as targeting.

A related but distinct direction is the use of whole cellular organelles as drug carriers. This line of work expands the concept of natural carriers beyond the more familiar extracellular vesicle model and shows that intracellular structures such as lipid droplets, lysosomes, and mitochondria may also be repurposed for delivery [69]. This shift is important because it suggests that drug delivery can draw not only from natural extracellular interfaces, but also from intracellular architectures that already possess specialized compartmentalization and transport functions. The value of these systems therefore lies in the close coupling between structure and function that has already been established in biology.

Ferritin adds another level of significance to this category because it represents a naturally assembled protein nanocage with built-in loading logic. Related studies have shown that its disassembly and reassembly behavior is directly relevant to payload loading and release, which links natural geometry to a controllable carrier design [80]. A similar logic appears in biomimetic metal organic frameworks, where biologically inspired structural principles are translated into engineered platforms for delivery and other biological applications [81]. Plant-derived polysaccharide-based carrier systems further support the broader idea that natural architectures can be converted into practical design rules for stabilization and transport [82]. Taken together, these examples show that natural geometry is no longer just a descriptive concept in biomimicry. It has become a practical foundation for rational carrier engineering. Most of these systems have not yet been validated in the bladder or upper urinary tract models and should therefore be regarded as general bioinspired carrier concepts rather than urinary-specific evidence.

Overall, the importance of natural carriers goes well beyond their biological origin. Their structural organization, compartmentalization, and interfacial properties all contribute directly to delivery performance. Future studies still need to improve isolation efficiency, engineering control, and large-scale production, but the central idea is already clear. Nature-derived systems provide a richer starting point for drug delivery design than many conventional synthetic platforms [69,81].

### 3.2. Motility Biomimicry: The “Artificial Swimmer”

Whereas natural carriers mainly highlight structural inheritance, motility biomimicry focuses on the role of active behavior in delivery. Passive delivery systems often provide limited control over where therapeutic cargo ultimately accumulates, and this limitation becomes more obvious in flowing biological environments or in anatomical regions that are difficult to access [55]. Motility biomimicry addresses this problem by introducing active movement into the delivery process (Figure 3) [83,84]. In recent years, the field has moved beyond conceptual discussions of microswimmers and has shifted toward more application-oriented questions, including actuation, cargo loading, navigation [85], imaging, and clinical translation [86,87]. In the context of intravesical therapy, however, the key issue is not simply whether a device can move, but whether active motion improves the delivery of a defined therapeutic cargo under bladder-specific washout conditions.

The current work commonly divides artificial swimmers into magnetic, anchored, self-propelled, and biohybrid systems. This classification matters because it shows that active delivery is no longer being treated as a single technology. Instead, it has become a broader design space in which different systems are matched to different biological barriers, flow conditions, and therapeutic tasks [87]. For this reason, the key question in this section is not simply whether a device can move, but whether that movement can meaningfully improve delivery performance in a controlled and medically relevant way. This point is especially important for intravesical treatment, because chemotherapeutics, immunotherapeutic agents, and radionuclide systems do not require the same degree of localization, penetration, or release control.

Among the available actuation strategies, magnetic control remains one of the most practical routes for in vivo use. Recent reviews emphasize that micrometer-scale magnetic microrobots are especially attractive for cargo delivery because they combine navigability with a greater loading capacity than many smaller nanoscale systems [88]. This translational relevance is now supported by direct bladder cancer evidence. Jia et al. recently developed magnetic-driven hydrogel robots loaded with mitomycin and showed that active magnetic guidance could be coupled to local chemotherapy in bladder cancer [89]. The importance of this study is not only that the system moves, but that it addresses a clinically familiar cargo and links magnetic localization to a specific intravesical therapeutic task. In this sense, magnetic propulsion becomes more convincing when discussed together with the drug class being delivered, rather than as a standalone engineering feature.

Bladder-specific evidence also shows that active motility is not limited to chemotherapy delivery. Simó et al. used urease-powered nanobots for radionuclide bladder cancer therapy in an orthotopic mouse model and observed enhanced tumor accumulation together with substantial tumor reduction [90]. Choi et al. extended the same general concept toward immunotherapy by developing a urease-powered nanomotor containing a STING agonist for bladder cancer immunotherapy [91]. Considered together, these studies indicate that the rationale for motility depends on the cargo class. For radionuclide systems, the main need is an improved localization and tissue coverage. For immunotherapy, active motion also serves to enhance local exposure and tumor penetration under washout conditions.

At the same time, clinical translation has become a central concern. Roadmap-style analyses now make it clear that mobile microrobotics is promising, but still faces substantial barriers before medical implementation becomes realistic [86]. These barriers include reliable imaging in vivo, stable control in complex biological environments, material safety, biodegradability, scalable fabrication, and regulatory feasibility. Much of the broader microrobotics literature remains non-urologic and is therefore most useful in defining control, imaging, manufacturing, and biodegradability problems rather than serving as direct proof for bladder cancer therapy. Bioinspired 4D transdermal microneedles reflect the same broader tendency in adaptive delivery design, even though they are not swimmers in the strict sense. Their value lies in showing how dynamic and responsive structures may improve localized therapy and patient compliance. In situ cellular hitchhiking also supports the broader principle that active or assisted transport can improve delivery behavior in complex in vivo environments [76]. This perspective is important because it repositions artificial swimmers from futuristic concepts to delivery systems that must eventually satisfy practical medical standards.

Taken together, motility biomimicry has become a substantive direction in active drug delivery rather than a speculative extension of nanomedicine. Its significance in urinary oncology is strongest when movement is tied to a defined therapeutic task, such as mitomycin delivery, STING-agonist-based immunotherapy [91], or radionuclide [90] localization in orthotopic bladder cancer models. The next phase of progress will likely depend on whether movement, imaging, local release, and biodegradability can be integrated within a single controllable platform. Only when these elements are addressed together will artificial swimmers begin to move from proof-of-concept devices toward therapeutically meaningful systems.

### 3.3. Surface Biomimicry: Antifouling and Retention

If the previous two sections emphasize structural inheritance and active motion, surface biomimicry focuses on how materials behave at the biological interface. Surface fouling remains a persistent problem for biomedical materials and devices because it can lead to protein adsorption, microbial accumulation, thrombosis-related events, or overall functional deterioration [92]. Surface biomimicry seeks to address this problem by borrowing design principles from natural interfaces that already resist contamination, promote self-cleaning, or perform different tasks on different sides of the same material [92,93]. The recent work on slippery-liquid-infused porous surfaces [94], biomimetic interfaces [95], and Janus hydrogels [96] shows that this field is shifting from simple antifouling coatings toward multifunctional interface engineering. In the urinary tract, this issue has a dual meaning, because a useful intravesical system must resist nonspecific fouling and premature loss while still allowing selective retention at the diseased urothelium.

One representative direction is the development of slippery-liquid-infused porous surfaces inspired by the pitcher plant. These interfaces exhibit smooth surfaces and very-low-contact-angle hysteresis, and they have shown clear promise in antibacterial, antithrombotic, and self-cleaning applications [94]. The significance of this work lies in the fact that it shows antifouling performance does not have to rely solely on chemical inertness. It can also be achieved through interfacial physics and lubricant stabilization. In this sense, biomimetic antifouling is not a single method, but a broader family of interface control strategies. For urinary applications, the relevance of this concept lies less in simple self-cleaning and more in whether interfacial control can reduce nonspecific adsorption or premature washout without compromising local therapeutic contact.

A more systematic perspective comes from recent reviews that connect microstructure, surface chemistry, and functional regulation within one framework [95]. This development is especially important because it moves the field beyond a descriptive imitation of natural surfaces and toward a more operational engineering language [48]. Janus hydrogels extend this discussion further by showing that asymmetric biomimetic interfaces can simultaneously support tissue integration on one side and reduce unwanted adhesion or contamination on the other [96]. Together, these studies indicate that retention and antifouling are not inherently contradictory. Instead, they can be spatially separated and optimized within the same material platform, which is particularly relevant for intravesical systems where excessive nonspecific adhesion may aggravate local irritation, whereas insufficient surface residence may lead to rapid washout before meaningful drug transfer occurs.

Related examples, such as seaweed-inspired anti-oil-fouling coatings and micro- and nanostructured antibacterial surfaces, further show that natural interfaces are valuable not because they merely resemble nature, but because their structural hierarchy and chemical properties are closely linked to function [97,98]. What makes these systems worth copying is the clear coupling between interface design and performance. Studies on multifunctional biomimetic coatings and mussel-inspired switchable superhydrophobic surfaces also support the idea that adhesive chemistry and fouling resistance can be jointly engineered rather than treated as competing properties [99,100]. Most of these studies are best interpreted as interfacial design inspiration rather than direct urinary-oncology evidence, unless they are tested under bladder-relevant conditions. In this sense, the higher goal of surface biomimicry is not simply to reduce attachment, but to control where and when attachment should occur.

Overall, surface biomimicry is moving from single-function coatings toward a multifunctional interface design. In urinary oncology, its translational relevance will depend on whether surface-engineered systems can balance antifouling behavior, selective retention, local tolerance, and delivery efficiency under repeated washout conditions. Future progress will still depend on greater durability, stronger long-term performance in complex in vivo environments, and scalable fabrication methods. Even so, the field already offers a clear principle for biomaterials design: natural surfaces do not only provide specific antifouling motifs, but also reveal how adhesion, lubrication, protection, and retention can be balanced within a single engineered system. The applications of structural and functional biomimetic innovations in drug delivery have been summarized in Table 1.

## 4. Bio-Inspired Evaluation and Precision Oncology

### 4.1. LOC-Based Evaluation of Biomimetic Delivery Strategies

**Figure 4:**
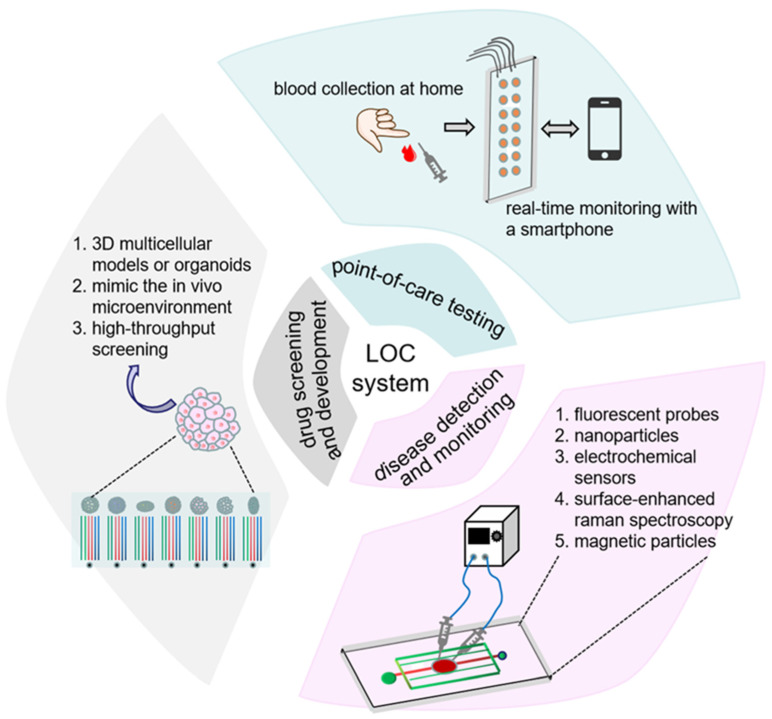
The integration of microfluidic system and various technologies develop its applications in biomedical field, such as advancement in drug discovery, point-of-care testing, and early disease diagnosis and monitoring.

The test of the bimomimetic drug delivery strategies is a high-expenditure-on-time-and-cost process. Fortunately, the development of a lab-on-a-chip (LOC) system, also termed micro total analytical system (μTAS), speeds up this process. This LOC system realizes laboratory bench-work on a small scale, and integrates the biomimetic material assembly, sample treatment, chemical reaction, and data process into a chip, which allows for the better evaluation of drug efficacy and a greater reduction in the total cost and duration [106]. The first bio-LOC system microfluidic device was developed in 1990s, which allows us to separate amino acids using capillary electrophoresis with up to 75,000 in about 15 s [107], greatly improving sample handling and separation. This breakthrough attracts more researchers to LOC development in the biomedicine field (Figure 4).

For example, in the drug discovery process, the LOC system enables us to automatically analyze and screen multiple compounds with various concentrations simultaneously, reducing the total cost and duration. Schuter et al. developed a robust and streamlined automated microfluidic three-dimensional culture platform that provides dynamic and combinatorial drug screens of tumor organoids [108]. This system controls culture conditions and screens drug efficacy in real time, and allows thousands of drug evaluations at once. Point-of-care testing is a new translation of LOC application. A combination of medical tests onto a microfluidic chip and its portable and inexpensive characteristics allow field testing or self-testing at home [109]. Coronavirus sickness 2019 (COVID-19) is a deadly disease, inducing acute respiratory distress syndrome and excessive mortality [110]. The prompt diagnosis of severe acute respiratory syndrome coronavirus 2 (SARS-CoV-2) infection is crucial for preventing the infection from spreading and determining the patient outcome. Sawank et al., developed a microfluidic system using nanoimmunoassay to detect anti-SARS-CoV-2 antibodies, which allows us to test 1024 blood samples at once. This system achieved a 100% specificity and 98% sensitivity, respectively, which is higher than the commercial enzyme-linked immunosorbent assay (ELISA). An ultralow-volume of 0.6 μL of whole blood and a simple sample collection with a finger prick make it suitable for a large-scale, low-cost, and widely accessible testing platform [111]. The early disease detection and biomarker monitoring have also been evolved by the LOC system. Studies showed that early detection and treatment for urinary cancer could improve the 5-year survival rate of 70–94% in renal cancer [112], and decrease by up to 49% the mortality rate in prostate cancer [113], which provide substantial benefits for patients with urinary cancer. The integration of the microfluidic system with various detection technologies, such as fluorescent probe detecting cancer biomarkers [114], nanoparticles achieving tumor detection and gene silencing [115], electrochemical sensors utilizing an electrochemical signal to detect circulating melanoma cells [116], surface-enhanced Raman spectroscopy (SERS) amplifying Raman signals to achieve the ultra-high sensitivity recognition of tumor-associated molecules [117], and magnetic particles for the isolation and concentration of target biomarkers, especially cancer markers [118], develops microfluidic biosensors, providing early precise biomarker detection with a high sensitivity and the continuous monitoring of cancer progression.

Specifically, the LOC system displays unique advantages in biomimetic material screening. For example, Song et al. established a multichannel microfluidic chip to screen the optimum cell membrane with a high tumor tropism [119]. Arduino et al. designed a novel production process of the biomimetic drug delivery system, showing a rapid and controlled production approach of biomimetic nanoparticles and enhanced homotypic targeting of metastatic melanoma cells [120]. Therefore, the LOC platform enables us to individually evaluate the function and therapeutic efficacy of the biomimetic-material-based drug system, achieving the high-throughput screening of urinary cancer therapeutics.

### 4.2. PDOs: The Bridge of Precision Oncology

To replicate the complex physiological structure and diverse cellular characteristics, it is essential to integrate the urinary cancer model into microfluidic devices for the large-scale evaluation of biomimetic-material-based drug efficacy, such as precise targeting, drug retention properties, and therapeutic responses. Three-dimensional tumor organoids, also referred to as ‘organ in a dish’, can be developed from self-organizing pluripotent stem cells, including embryonic stem cells and patient-derived stem cells. They can replicate the complex pathophysiogical structure, cellular compositions, and heterogeneity of the original tumor [121,122], and the tumor microenvironment, including an acidic pH, hypoxia, and excess ROS and GSH [121]. These 3D tumor organoids make it possible to assess the drug efficacy, delivery strategy feasibility, and therapeutic targeting, and even achieve personalized therapy for patient-derived tumor organoids (PDOs). For example, PDOs from pancreatic ductal adenocarcinoma were used to evaluate an epigenetic drug UNC1999. This drug showed significant therapeutic effects in three of five PDOs, in which these three PDOs retained the epigenetic signature of primary tumor [123]. He et al. developed a PDO platform to assess patient-specific drug sensitivity in metastatic colorectal cancer. This platform successfully tested chemosensitivity (5-fluorouracil, oxaliplatin, and irinoteca) in 42 organoids [124], providing early predictions for clinical therapeutic efficacy. Furthermore, supplementing organoids with cellular components, such as stromal cells, vascular endothelial cells, immune cells, and ECM, can more closely mimic the in vivo tumor microenvironment [125]. For example, Takeuchi et al. created a fused pancreatic cancer organoid through the co-culture of patient-derived pancreatic ductal adenocarcinoma cells with mesenchymal and vascular endothelial cells. Additionally, these organoids could be further induced to form two distinct types of pancreatic ductal adenocarcinoma (quiescent and proliferation organoids). The anticancer drug (e.g., gemcitabine, 5-FU, and paclitaxel) resistance screening was performed and showed different responses in these two organoids [126]. This multicellular organoid not only replicates the complex tumor microenvironment but also flexibly resembles tumor heterogeneity, offering a potential platform for screening anticancer drugs. To mimic the tumor immune microenvironment, Neal et al. developed PDOs in syngeneic immunocompetent hosts where immune cells (T cells, B cells, natural killer cells, and macrophages) infiltrated PDOs. This model preserved the immune properties of the original tumor tissues and recapitulated the PD-1-dependent immune checkpoint, providing a minimal system with which to establish the in vitro immunotherapy model and evaluate the therapeutic responses [127]. Therefore, multicellular PDOs can be used as a valuable tool with which to predict the drug efficacy and clinical outcomes, promisingly guiding personalized therapy.

**Figure 5:**
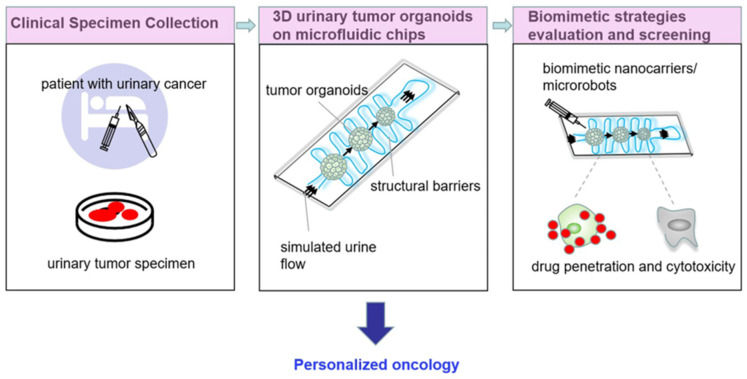
Schematic illustration of the personalized oncology for urinary cancer therapeutics. First, clinical tumor tissues are collected from the patient with urinary cancer. Subsequently, we have the 3D Organoid-on-a-Chip Culture: harvested cells are cultured to form tumor organoids within a microfluidic platform, which effectively recapitulates the in vivo tumor microenvironment, including the biological barrier and simulated urine washout effect. Then, high-throughput screening and evaluation are performed: diverse biomimetic nanocarriers and microrobots are introduced into the chip system. Finally, personalized therapeutic strategy is designed.

Therefore, the integration of the urinary cancer 3D tumor model or organoids in a microfluidic chip can reconstitute a urinary microenvironment that mimics the in vivo tumor pathophysiological process, offering a reliable way for the development of precision oncology. For example, Tak et al. developed a 3D microfluidic chip with a multi-cell co-culture, recreating the bladder cancer model with different morphological characteristics for the establishment of anticancer drug resistance among four differential cell levels [128]. This platform helps to predict drug responses and further provides an appropriate treatment for bladder cancer patients. To recapitulate the inherent features including the complex structural and biological composition and diversity functions of the primary tumor, Viergever et al. generated urine-derived bladder cancer organoids. These organoids were shown to be highly similar to the original patient tumors in terms of single-nucleotide polymorphisms (92.56%) and insertions and deletions (91.54%) [129]. This tumor organoid system makes it possible to longitudinally monitor the drug response and even guides the personal choice for second-line therapy. Similarly, Minoli et al. established PDOs from different bladder cancer stages and grades, which preserved the genetic heterogeneity of the original tumors. These PDOs were used to test drug screenings, the standard of care, and FDA-approved compounds, eventually determining the degree of alignment between bladder cancer evolution and drug responses [130]. Therefore, PDOs can effectively replicate the histopathological and genetic heterogeneity of urinary cancers that are very representative of clinical cases. This model combined with the LOC system potentially allows us to assess the biomimetic drug delivery strategies and predict treatment outcomes for precision oncology (Figure 5).

## 5. Challenges and Translation

Bio-hybrid systems, especially at the nano scale, endow enhanced performance in drug delivery, tissue engineering, early diagnosis, therapeutics, and even complex perception and movement function through the integration of biomimicry, advanced nanotechnologies, chemical engineering, AI, and an artificial nervous system. These potential applications contribute to the advancement of the future medical field, ultimately improving quality of life; however, the development and usage of biomimetic materials inevitably bring about new challenges, which hinder their wild application and clinical translation. The first issue is scalability: laboratory small-scale production inevitably presents a uniformity in size and composition, which is not conducive to mass production. Cost control is the second factor. The high expenses associated with biomimetic materials and the manufacturing process could limit clinical widespread application. While synthetic materials are easily scalable with a high consistency via the use of advanced synthesis technology [131], the mass production of biomimetic materials is limited by the high production cost, because they often incorporate natural components, such as cellular contents and biological enzymes, which are costly [132]. Furthermore, the maintenance of the activity of these biological components requires specialized facilities and a controlled storage environment, which significantly increases the overall production cost [133].

The translation of biomimetic nanosystems faces significant uniformity and scalability hurdles. At the laboratory scale, biomimetic engineering can precisely emulate natural biological structures and functions [134], successfully integrating properties such as spore-inspired durability [135], mussel-derived wet adhesion [136], and viral-mediated tissue penetration [137]. However, the increasing complexity of these systems complicates mass production. For instance, while highly customized platforms, such as tumor-cell-membrane-camouflaged nanoparticles, have demonstrated excellent preclinical efficacy in non-urologic models like glioblastoma [138], scaling up such delicate biological components for intravesical therapy remains economically and technically daunting. Furthermore, while advanced fabrication technologies, including droplet microfluidics [139] and 3D printing [140], allow for the precise control of nanoparticle sizes, shapes, and drug encapsulation, they primarily serve as excellent prototyping and screening tools; they do not, by themselves, resolve the industrial-scale manufacturing bottlenecks for complex bio-hybrids. To address these high-cost concerns, exploring easily accessible biomimetic templates, such as spike-like architectural pollen and pitcher plant-inspired slippery surfaces, coupled with cost-effective fabrication methods will be essential for enhancing the true scalability for clinical applications in urinary oncology.

In addition to the referred limits in uniformity and reproducibility in size and function, and production cost control, biohybrid translation from the laboratory to clinical applications also faces biohybrid classification and biological safety challenges in urinary cancer theranostic strategies.

### 5.1. Regulatory Hurdles: How to Classify “Bio-Hybrid” Devices (Drug vs. Device vs. Biologic)

The bio-hybrid system integrates biomimetic material, anti-cancer drugs, and/or living cells to obtain a better cancer therapeutic efficacy, such as the fish structural biomimetic microrobot-mediated multidrug delivery system [141], bacteria-enhanced intratumoral drug transport [142], and macrophage template-based microrobots for targeted drug delivery [143]. Therefore, in terms of drug delivery function, it belongs to the drug concept; as advanced biomimetic materials, they are sorted into the device category; the incorporation of living cells causes it to be classified in the biological field. However, these classifications have completely different quality specifications and ethical concerns. The usage of biological components requires us to fully consider the bio-safety and ethical issues. For example, microbial and viral biohybrid deployment comes with a real-time concern of bacterial mutation and gene exposure risk, which demands operators strictly follow the experimental procedure and perform all works in the biosafety cabinet. The use of animal subjects has to abide by the 4R principles including reduction, replacement, refinement, and reproducibility [144], aiming to maximize the reduction in the need for animal testing. Therefore, the effective regulation of bio-hybrid system requires us to define its source of functions and specific category. Fortunately, with the development of the bio-hybrid system, the regulatory landscape is gradually adopting a more flexible, risk-based, and harmonized approach [145,146]. Despite the fact that it accelerates the development and clinical transformation of bio-hybrid produce, the entire process from design through post-market is required to be strictly regulated.

### 5.2. Safety: Long-Term Fate of Magnetic Materials and Biological Carriers in the Urinary Tract

While bio-hybrid design leverages the natural structure, component, and biological movement capability, the inherent nature of the biomimetic system, such as the magnetic properties of nanomaterials and biological carriers with a spore-like surface architecture, means it generally increases the bio-safety concern in the urinary tract. A normal urinary tract is covered by umbrella cells in an organically and densely arrangement pattern, which form a contiguous hollow system that defends against microbial infection and supports urine collection, transport, storage, and expulsion [20]. Under disease conditions such as a tumor, the urinary microenvironment will be remodeled, resulting in its volatility in environmental factors. Despite the magnetic properties providing precise targeting and hyperthermia therapy at the tumor site under an alternating magnetic field [147], the accumulation and toxicity of intrinsic metal components such as Fe, Co, Mn, Zn, N, and/or O elements may impair the structure and function of the urinary tract. In addition, a substantial amount of iron ions is prone to generating excessive reactive oxygen species in a biological environment, which exhausts the ascorbate and glutathione levels. This oxidative stress impairs cellular-redox-regulated functions, leading to DNA damages, cytotoxicity, and the apoptosis of urothelial cells [148]. Therefore, magnetic material application must fully consider the concentration, components, and clearance or retrieval-related issues.

Biomimetic nanocarriers offer a targeted specificity and prolonged drug dwell time. However, degradation and clearance issues need to be considered. Unlike systemically injected nanoparticles, which are eliminated via renal excretion, hepatobiliary elimination, and mononuclear phagocyte system clearance [149], intravesically instilled biomimetic nanocarriers are primarily degraded through periodic voiding. For those penetrating into the tumor sites in a targeted manner, they are reabsorbed into the blood circulation and subsequently eliminated through the kidneys [150]. Therefore, bio-hybrid design needs to achieve a dynamic balance between “on-demand drug concentrations and safe degradative and metabolic trajectories“ in the urinary system, not only overcoming the rapid clearance-induced compromised therapeutic efficacy but also avoiding the sluggishly degradative process-associated cytotoxicity.

## 6. Conclusions

The unique anatomical structure characterized by a superficial GAG layer and specialized umbrella cells that prevent bacteria from sticking and protect the underlying tissue from the highly acidic and toxic components of urine [151], and the physiological function properties of collection, transport, storage, and voiding, facilitating high-velocity excretion with peak flow rates typically ranging from 15 to 30 mL/s [152] limit the penetration, retention, and spread of a drug for urinary cancer treatment. The exploration of biomimicry and the development of advanced nanotechnology closely mimicking the natural structure and functionalities have marked significant milestones in the biomedical field. These biomimetic strategies offer a new promise to solve the “mechanical” and “biological” barriers of urinary cancer therapeutics. The integration of the natural structure and component, which could maximally evade the washout effect and immune-clearance effects, into nanocarriers makes it possible to deliver the relevant drugs to urinary cancer tissue and potentially paves the way for the development of biomimetic drug delivery, diagnosis, and therapeutics systems. Therefore, this may provide a new strategy for urinary cancer theranostics.

In the future, sustained interdisciplinary cooperation is essential in order to overcome the existing challenges. In addition, improving the technologies and functionality of the biomimetic system, such as the development of microrobots and the integration of cell membranes or immunotherapy, enables us to enhance its biocompatibility, accurate targeting, and therapeutic synergy. The convergence of nanotechnology-engineered motility, and biological intelligence promisingly evolves the therapeutic patterns of urinary cancer, offering a more precise and efficient theranostic strategy.

## Figures and Tables

**Table 1:** Summary of representative methods for structural and functional biomimetic drug delivery.

Method	Principle	Urinary Oncology Relevance	Intended Therapeutic Cargo	Evidence Level	Major Translation Barriers in the Urinary Tract	Advantages	Limitations
Extracellular-vesicle-based carriers [79,101]	Natural membrane vesicles are used to deliver proteins, nucleic acids, or small molecules	Moderate. Potentially relevant to intravesical uptake and cargo protection, but direct bladder cancer validation remains limited in this section.	Proteins, nucleic acids, small molecules	Moderate	Urine dilution, batch heterogeneity, limited bladder specific validation	High biocompatibility, low immunogenicity, efficient cellular uptake, and good cargo protection	Limited isolation efficiency, compositional heterogeneity, and difficulties in large-scale production and engineering
Organelle-based carriers [69]	Natural intracellular compartments such as lipid droplets, lysosomes, or mitochondria are repurposed for drug delivery	Low to moderate. Conceptually attractive, but urinary-specific validation is limited.	Proteins, nucleic acids, specialized intracellular cargo	Low	Instability in urine, poor standardization, uncertain urothelial compatibility, scale-up difficulty	Unique biological functions and potential for specialized intracellular delivery	Complex preparation, poor standardization, and limited stability and scalability
Ferritin nanocage carriers [80,102]	Ferritin protein cages are used as natural nanocontainers for cargo loading and release	Moderate as a general carrier concept, but limited direct intravesical evidence.	Small molecules, imaging probes	Low	Limited mucosal retention, uncertain GAG penetration, restricted bladder-specific evidence	Well-defined structure, good stability, and tunable loading strategy	Restricted cargo types and limited delivery efficiency
Tumor selective mucoadhesive hydrogels [58]	Mucoadhesive systems are engineered to retain cargo locally while promoting selective bladder tumor contact	High. Directly relevant to intravesical chemotherapy and local retention design.	Intravesical chemotherapy	High	Selective gelation, post-treatment clearance, local urothelial tolerance, material degradation	Improved local dwell time, selective local activity, clinically relevant intravesical design	Need to balance retention with clearance and irritation risk
Virus-inspired and mucus-penetrating intravesical nanocarriers [64]	Biomimetic nanocarriers mimic viral penetration logic and mucus traversal to improve delivery across bladder barriers	High. Direct orthotopic bladder cancer evidence is available.	Intravesical chemotherapy, photosensitizers	High	GAG penetration, tumor selectivity, off-target urothelial uptake, local tolerance	Enhanced mucus penetration, improved tumor targeting, better local efficacy	Complex design and barrier-dependent performance
Engineered membrane-camouflaged systems [72]	Cell membrane coating or membrane engineering is used to improve immune evasion and targeting	High. Bladder-specific-immunotherapy-oriented examples are now available.	Immune agonists, photothermal immunotherapy payloads, targeted nanomedicine	High	Membrane standardization, immune safety, manufacturing complexity, long-term reproducibility	Reduced immune clearance, programmable targeting, improved bladder-specific relevance	Complex preparation, quality control challenges, limited translational standardization
Magnetic microrobots [77]	External magnetic fields guide microscale carriers for active transport and localized release	High. Directly relevant to intravesical localization and active-tumor-directed delivery.	Chemotherapeutics, immune agonists, radionuclide payloads	High	Imaging guidance, control under urine flow, biodegradability, metal safety, retention without obstruction	Precise navigation, active transport, and high potential for localized therapy	Dependence on external control and imaging systems, with unresolved biodegradability and safety issues
Self-propelled and biohybrid swimmers [90]	Autonomous propulsion or biologically assisted motion is introduced to enhance transport and local accumulation	Moderate to high. Strong conceptual value with emerging bladder-specific systems.	Chemotherapeutics, immunotherapeutic agents, imaging or radionuclide cargo	Moderate to high	Fuel compatibility in urine, controllability, biosafety, clearance after treatment	Active motion, strong penetration potential, flexible design	Limited in vivo controllability, complex fabrication, safety concerns
Bioinspired 4D microneedles and adaptive devices [83,103]	Stimuli-responsive structures change shape or function over time to improve delivery performance	Low for direct urinary oncology, mainly conceptual inspiration for adaptive local delivery.	Local-release drugs, small molecules, biologics	Low	Anatomical mismatch with urinary tract deployment, retention control, material reliability in wet lumen	Strong adaptability and improved local delivery efficiency	Complex design and insufficient long-term reliability and manufacturing consistency
Slippery-liquid-infused porous surfaces [94,104]	Lubricant-stabilized interfaces reduce adhesion of contaminants, bacteria, or blood components	Moderate as a surface engineering concept for urinary devices and interfaces, but limited direct tumor delivery evidence.	Not primarily a cargo carrier. Better viewed as an interfacial support strategy	Low	Lubricant loss, repeated voiding durability, long-term urothelial tolerance	Excellent antifouling, antibacterial, and antithrombotic performance	Limited long-term lubricant stability and interfacial durability
Janus hydrogel interfaces [96]	Asymmetric interfaces are designed to provide distinct functions on opposite sides	Moderate. Potentially relevant to combining retention and antifouling in bladder applications.	Local-release drugs, small molecules, interface active systems	Low to moderate	Directional deployment in bladder, local irritation, structural stability during filling and voiding	Can integrate tissue adhesion and antifouling functions within one platform	Relatively complex fabrication and limited structural stability
Multifunctional biomimetic coatings [48,105]	Surface chemistry and microstructure are jointly engineered to integrate multiple interfacial functions	Moderate. Useful for local retention and interface control, but most examples remain non-urinary.	Surface-bound drugs, antibacterial agents, local-release coatings	Low	Balancing retention with irritation, coating durability, reproducibility in urine	High tunability and broad utility in biomedical interface design.	Complex parameter optimization and limited scalability and long-term stability

## Data Availability

No new data were created or analyzed in this study. Data sharing is not applicable to this article.
